# Cingulate NMDA NR2B receptors contribute to morphine-induced analgesic tolerance

**DOI:** 10.1186/1756-6606-1-2

**Published:** 2008-06-17

**Authors:** Shanelle W Ko, Long-Jun Wu, Fanny Shum, Jessica Quan, Min Zhuo

**Affiliations:** 1 Department of Physiology, Faculty of Medicine, University of Toronto, University of Toronto Centre for the Study of Pain, 1 King's College Circle, Toronto, M5S 1A8, Canada

## Abstract

Morphine is widely used to treat chronic pain, however its utility is hindered by the development of tolerance to its analgesic effects. While N-methyl-D-aspartate (NMDA) receptors are known to play roles in morphine tolerance and dependence, less is known about the roles of individual NMDA receptor subtypes. In this study, Ro 256981, an antagonist of the NMDA receptor subunit NR2B, was used to reduce the expression of analgesic tolerance to morphine. The mechanisms altered with chronic drug use share similarities with those underlying the establishment of long-tem potentiation (LTP) and behavioral memory. Since NMDA NR2B receptors in the anterior cingulate cortex (ACC) play roles in the establishment of LTP and fear memory, we explored their role in changes that occur in this region after chronic morphine. Both systemic and intra-ACC inhibition of NR2B in morphine-tolerant animals inhibited the expression of analgesic tolerance. Electrophysiological recordings revealed a significant increase in the NR2B component of NMDA receptor mediated excitatory postsynaptic currents (EPSCs), at both synaptic and extra-synaptic sites. However, there was no change in alpha-amino-3-hydroxy-5-methyl-4-isoxazolepropionic acid (AMPA) receptor mediated EPSCs. This study suggests that selective inhibition of NMDA NR2B receptors may prove useful in combating the development of analgesic tolerance to morphine and proposes a novel role for the ACC in opioid tolerance and morphine induced changes in synaptic plasticity.

## Background

Although morphine is the most widely used analgesic to treat moderate to severe pain its use is hindered by the development of physical dependence and tolerance to its analgesic effects. The utility of N-methyl-D-aspartate receptor (NMDAR) antagonists in both potentiating and prolonging the analgesic effects of morphine while attenuating analgesic tolerance and physical withdrawal symptoms has been widely reported (see [1-5] for review). For example, the non-competitive NMDAR antagonist MK801 has been shown to modulate morphine analgesia, reverse analgesic tolerance and reduce withdrawal behaviors [6-9]. Pharmacological manipulation of NMDA receptor activity may pose a useful strategy for increasing the efficacy of morphine as a treatment for chronic pain in the future.

NMDARs are composed of NR1, NR2 (A, B, C, and D) and NR3 (A and B) subunits in the central nervous system. It is known that the NR2A and NR2B subunits predominate in the forebrain neurons, where they determine many functional properties of NMDARs [10,11]. For example, NR2B containing NMDA receptors desensitize less and take longer to recover from desensitization as compared to NR2A containing NMDA receptors [10,12]. The down regulation of NR2B expression throughout development is marked by a concomitant change in NMDA receptor function [13]. The unique functional properties of NR2B make it an attractive target for those who wish to study the mechanisms behind experience dependent changes in synaptic plasticity and behavioral responses.

The anterior cingulate cortex (ACC) plays important roles in emotion, addiction, learning and memory and persistent pain [14-17]. Overexpression of NR2B in the ACC and other forebrain regions significantly enhanced learning and memory as well as chronic pain caused by peripheral inflammation [18,19]. NR2B receptors expressed in the ACC also appear to play a role in synaptic plasticity (including LTP and long-term depression (LTD)) and the expression of fear memory [20,21]. Since addiction and memory share certain intracellular cascades in common [22,23], we wanted to determine if NR2B receptors in the ACC play a role in the expression of analgesic tolerance and changes in plasticity occurring after chronic morphine use. Our results reveal a significant enhancement of NMDA NR2B mediated responses in the ACC after chronic morphine treatment and suggest that such an enhancement may contribute to the development of morphine tolerance.

## Results

### NR2B plays a role in acute morphine-induced analgesia

**Figure 1:**
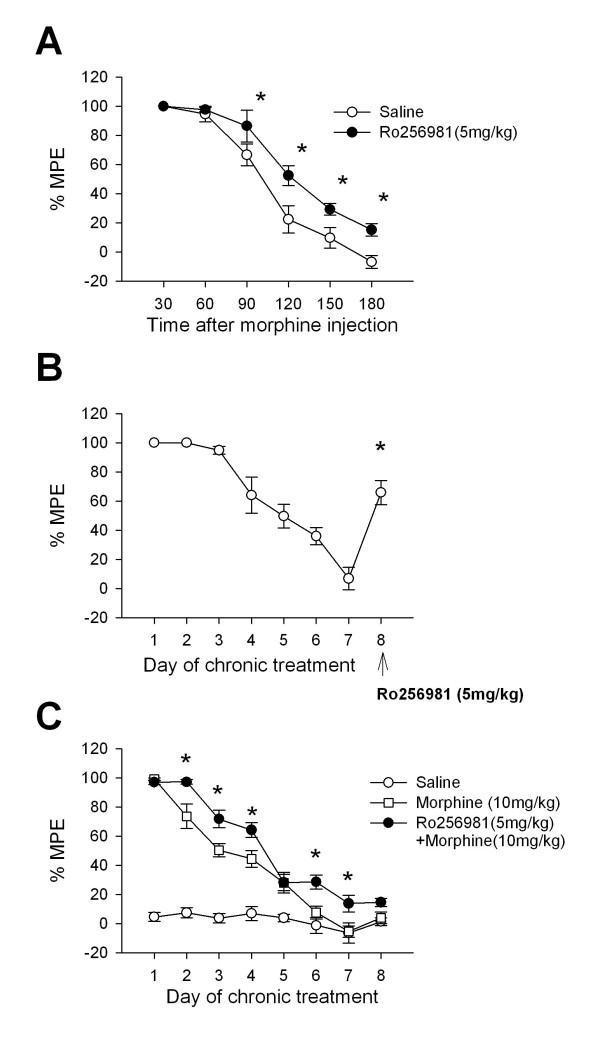
**The role of NR2B receptors in acute morphine-induced analgesia and analgesic tolerance**. (A) Morphine analgesia was greater in mice pretreated with Ro25-6981 (5 mg/kg, i.p., n = 5) compared to mice pretreated with saline (n = 4) beginning 90 min after injection. There was a significant effect of treatment (p < 0.05) with significant differences occurring at 90, 120, 150 and 180 minutes after injection (p < 0.05 for all). * represents a significant difference from saline injected mice. (B) Response latencies were significantly increased by Ro 256981 injection (5 mg/kg, i.p., n = 8). Day 7 vs. Day 8, p < 0.001). * represents a significant difference from responses on Day 6 and 7. (C) Daily co-administration of Ro 256981 (5 mg/kg, i.p.) with morphine (10 mg/kg, s.c.) attenuates opioid analgesic tolerance (p < 0.001) compared to mice receiving morphine (n = 7) or saline (n = 5). There was no difference between morphine treated and Ro256981+morphine treated mice on Day 1 (p = 0.74) but a significant difference arose on Day 2 (p < 0.001) and persisted during Days 3, 4, 6 and 7 (p < 0.001, p < 0.01, p < 0.05, p < 0.05 respectively).

NMDA receptor antagonists are reported to potentiate, inhibit, or not to alter morphine antinociception with variable results arising from the use of different doses of antagonist and morphine, as well as experimental animals and tests for nociception [1]. To determine the effect of Ro 256981 on acute morphine antinociception, mice were injected intraperitoneally with either Ro 256981 or saline (n = 4, i.p.) before receiving a single injection of morphine (10 mg/kg, s.c), and hot-plate response latencies were recorded every 30 min afterwards for three hours. Statistical analysis revealed a significant affect of treatment (p < 0.05) and time (p < 0.001) with a significant interaction between treatment and time (p < 0.05). Significant differences between response latencies occurred 90, 120, 150 and 180 minutes after morphine injection (p < 0.05 for all, Figure 1A). It is important to note that Ro 256981 is not in itself analgesic at this dose [21]. These results suggest that Ro 256981 potentiates the analgesic effect of morphine.

### NR2B inhibition affects opioid analgesic tolerance

To determine if antagonizing the activity of NR2B can affect opioid analgesic tolerance, mice (n = 8) were given eight daily injections of morphine (10 mg/kg, s.c.) and received Ro 256981 (5 mg/kg, i.p.) before the last morphine injection. One way repeated measures ANOVA revealed a significant affect of treatment (p < 0.001) with significant increases in response latency occurring between Day 8 (Ro + morphine) and Days 6 and 7 (p < 0.001, p < 0.05 respectively, morphine alone, Figure 1B). However, Ro 256981 did not totally reverse the established opioid tolerance since there were still significant differences between response latencies on Day 8, as compared to Day 1 (p < 0.01), Day 2 (p < 0.01) and Day 3 (p < 0.05). Interestingly, there was not a significant difference between responses on Day 8 and those on Days 4 and 5 (p = 1.00 and p = 0.61 respectively), suggesting that Ro 256981 administration on the 8^th ^day of morphine treatment restored morphine-induced antinociception to a level early on in the development of analgesic tolerance. Hot-plate responses were significantly longer on Days 4 and 5, as compared to Day 7 (p < 0.001 for both).

### Attenuation of analgesic tolerance by chronic co-administration of Ro 256981 with morphine

To determine if NR2B receptors play a role in the acquisition of opioid analgesic tolerance, Ro 256981 was co-administered daily with morphine. In this experiment, mice received either Ro 256981 (5 mg/kg, i.p., n = 11) before morphine (10 mg/kg, s.c.), morphine alone (n = 7) or saline (n = 5) daily for eight days. Statistical analysis revealed a significant affect of treatment (p < 0.001) and day (p < 0.001) as well as a significant interaction between treatment and day (p < 0.001, Figure 1C). Importantly, there were no significant differences across days for mice receiving daily saline injections. Tolerance developed in mice receiving morphine alone (Day 1 vs. Day 8: p < 0.001) and in mice receiving both Ro 256981 and morphine (Day 1 vs. Day 8: p < 0.001). However, post-hoc analysis revealed significant differences in response latencies between treatment groups on individual days. For example, there was no difference between morphine treated and Ro 256981 and morphine treated mice on Day 1 (p = 0.74), but a significant difference arose on Day 2 (p < 0.001) and persisted during Days 3, 4, 6 and 7 (p < 0.001, p < 0.01, p < 0.05, p < 0.05 respectively). It is also interesting to note that significant differences between morphine-treated mice and control mice receiving saline disappeared by the 6^th ^day of treatment (p = 0.28), which did not occur until the 8^th ^day in mice receiving both Ro 256981 and morphine (p = 0.17). Taken together, these results suggest that daily co-administration of Ro 256981 with morphine can hinder the development of opioid analgesic tolerance but not completely abolish its establishment, at least at the dose tested in this study.

### NR2B receptors in the ACC play a role in the expression of opioid tolerance

**Figure 2:**
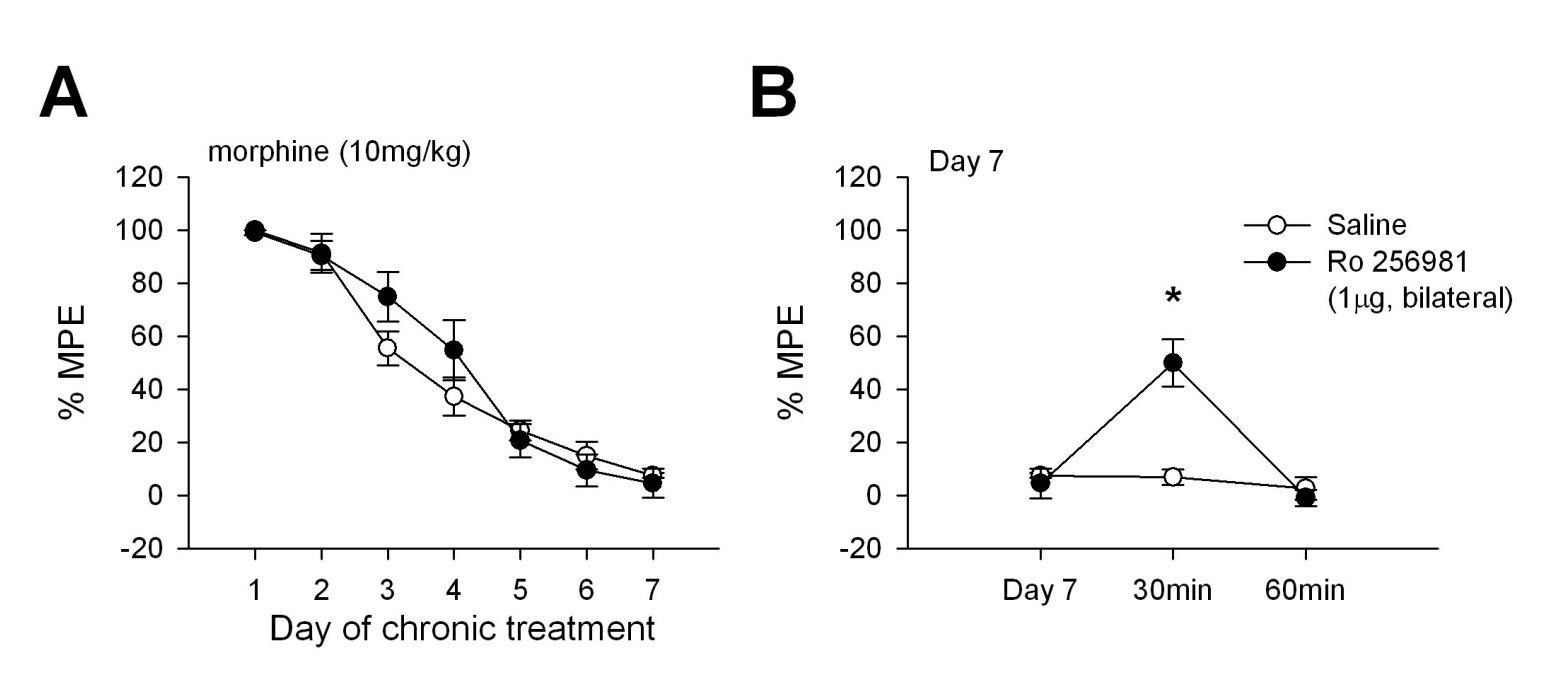
**Antagonism of NR2B receptors in the ACC can reverse opioid analgesic tolerance compared to mice receiving saline in the ACC**. (A) Analgesic tolerance in ACC cannulated mice developed over the seven-day test period. (B) Bilateral microinjection of Ro 256981 (1 μg in 1 μl, bilaterally, n = 7; filled circles) into the ACC significantly increased response latencies compared to mice receiving saline injections (n = 5; open circles) and to responses on Day 7.

To determine if inhibiting NR2B receptors in the ACC could reverse established analgesic tolerance, intra-ACC injections of Ro 256981 were performed in tolerant mice. Daily injections of morphine (10 mg/kg, s.c.) were administered to mice implanted bilaterally with cannulas directed to the ACC. Before testing hotplate response latencies on the eighth day, mice received either Ro 256981 (n = 7, 1 μg/μl, bilaterally) or saline (n = 5) injections in the ACC. While there were no differences in response latencies on the seventh day of testing between groups (p = 0.72), there was a significant difference in responses 30 minutes after microinjection (p < 0.001, Figure 2B). The effect of Ro 256981 in the ACC was abolished by one hour after microinjection (p = 0.66). These results indicate that NR2B receptors in the ACC play a role in the behavioral responses associated with opioid analgesic tolerance.

### AMPA receptor mediated synaptic transmission after morphine treatment

**Figure 3:**
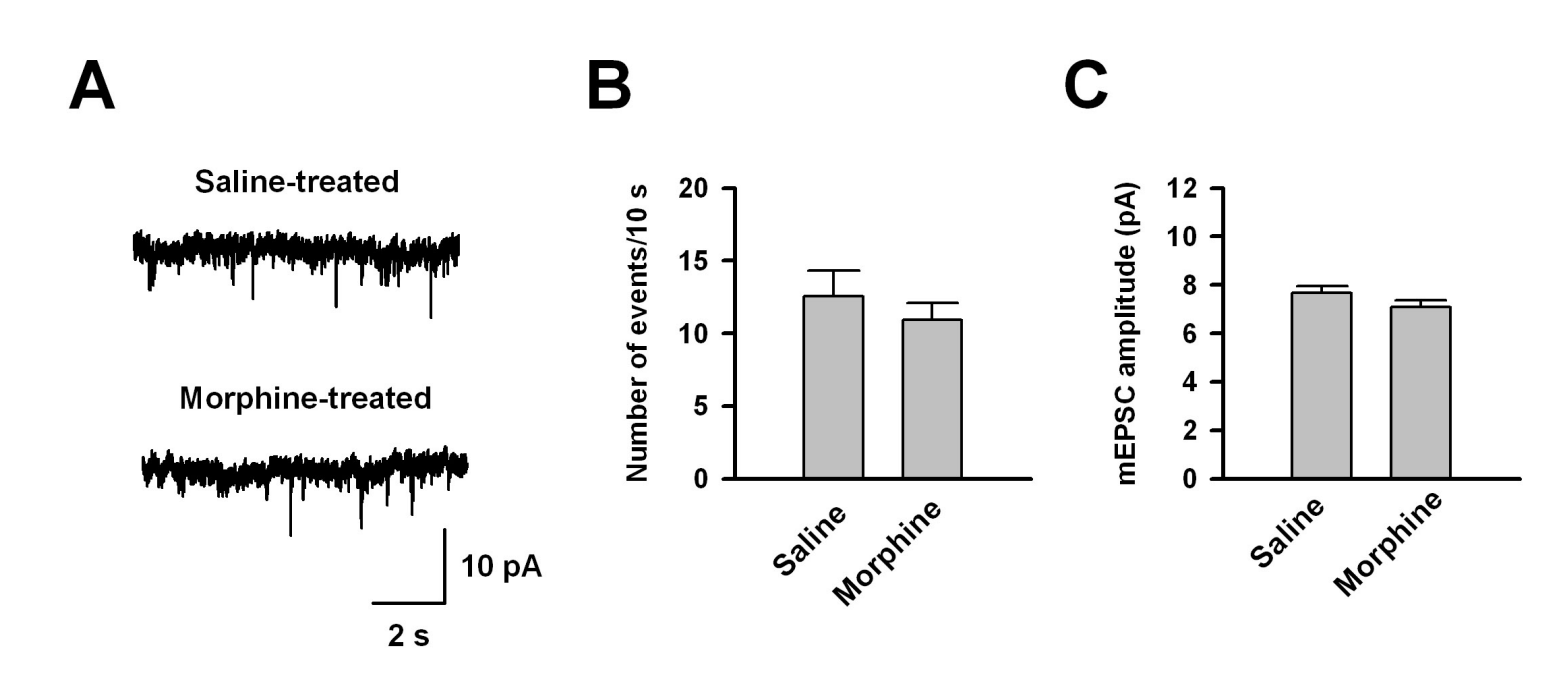
**No change in mEPSCs in morphine treated mice**. (A) Representative traces showing mEPSCs in saline (upper) or morphine treated mice (lower). (B) Pooled data showed that there is no difference in mEPSC frequency in saline (n = 16) and morphine treated groups (n = 16). (C) No difference in amplitude of mEPSCs in the ACC of saline (n = 16) and morphine treated groups (n = 16).

To determine whether synaptic transmission was altered in morphine tolerant mice, conventional whole-cell patch clamp recordings were performed in visually identified pyramidal neurons in layer II/III of the ACC. We recorded AMPA receptor-mediated miniature excitatory postsynaptic currents (mEPSCs) in saline or morphine-treated mice. In the presence of tetrodoxin (1 μM), AP5 (50 μM) and picrotoxin (100 μM), AMPA mEPSCs were pharmacologically isolated (Figure 3A). The frequency of mEPSCs in saline treated mice was 1.3 ± 0.2 Hz (n = 15). No significant difference was found in the frequency of mEPSCs in morphine treated mice (1.1 ± 0.1 Hz, n = 16, P = 0.48) compared to saline (Figure 3A and 3B). Likewise, there was no difference in mEPSC amplitude between saline treated (7.1 ± 0.3 pA, n = 16) and morphine treated mice (7.6 ± 0.3 pA, n = 16, P = 0.17) (Figure 3A and 3C). These results suggest that AMPA receptor-mediated synaptic transmission is normal in morphine tolerant mice.

### Functional upregulation of synaptic and extrasynaptic NR2B in morphine tolerant mice

**Figure 4:**
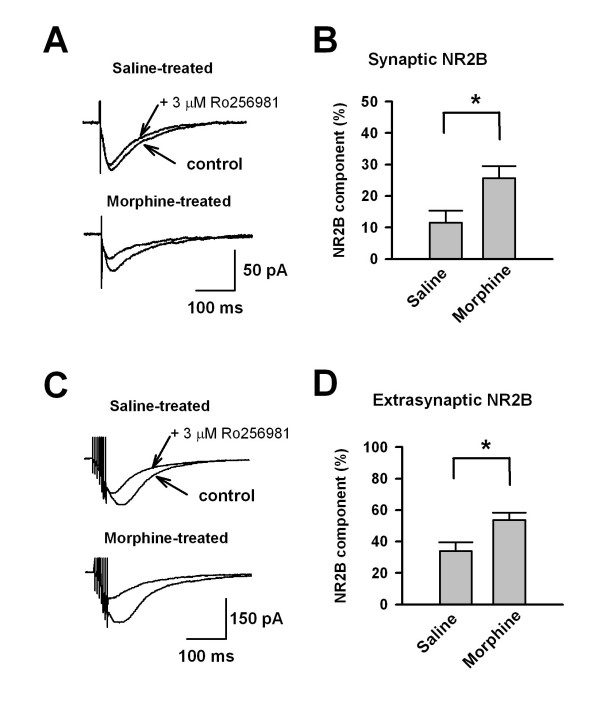
**Enhanced NR2B NMDA receptor function in morphine-treated mice**. (A) Representative traces showing that the NR2B component was revealed by application of Ro256981 (3 μM) in saline (upper) or morphine treated mice (lower). (B) Pooled data showed a significant increase in the NR2B component in morphine treated mice (n = 8) compared with that of saline group (n = 6). (C) A short train of stimuli (200 Hz, 7 pulses) induced lager NMDA EPSCs. The NR2B component, including extrasynaptic NR2B, was revealed by application of Ro 256981 (3 μM) in saline (upper) or morphine treated mice (lower). (D) Pooled data showed a significant increase in the NR2B component of short train-induced NMDA current in morphine treated mice (n = 7) compared with that of the saline group (n = 7).

We have previously reported roles for the NR2B subunit in synaptic plasticity in the ACC [20,21,24]. To determine if NR2B receptors in the ACC were affected by chronic morphine treatment, ACC neurons were held at a potential of -20 mV to relieve the Mg^2+ ^blockade and NMDA EPSCs were isolated in the presence of CNQX (50 μM) and picrotoxin (100 μM). Perfusion of Ro 256981 (3 μM) caused a substantial decrease in NMDA EPSCs (Figure 4A), referred to as the NR2B component. In saline treated mice, the NR2B component was 11.6 ± 3.8% of the total NMDA current (n = 6). A significant increase in the NR2B component was observed in morphine treated mice (25.6 ± 3.8% of total currents, n = 8, P < 0.05) (Figure 4A and 4B). Next, a short train of stimuli consisting of 7 pulses at 200 Hz was used to study the extrasynaptic NR2B component in the ACC after chronic morphine treatment. Consistent with previous studies in the hippocampus [25], this stimulation induced larger NMDA EPSCs with a slower decay (Figure 4C), suggesting that extrasynaptic NMDA receptors are involved. Ro 256981 (3 μM) blocked 34.1 ± 5.4% (n = 7) of the total train-induced NMDA current in saline treated mice (Figure 4C and 4D), while a significant increase in the NR2B component was again seen in morphine treated mice (53.7 ± 4.5%, n = 7, P < 0.05) (Figure 4C and 4D). Taken together, these results suggest that chronic morphine treatment increases both synaptic and extrasynaptic NR2B NMDA receptor activity.

### Enhanced LTP in the ACC of morphine treated mice

**Figure 5:**
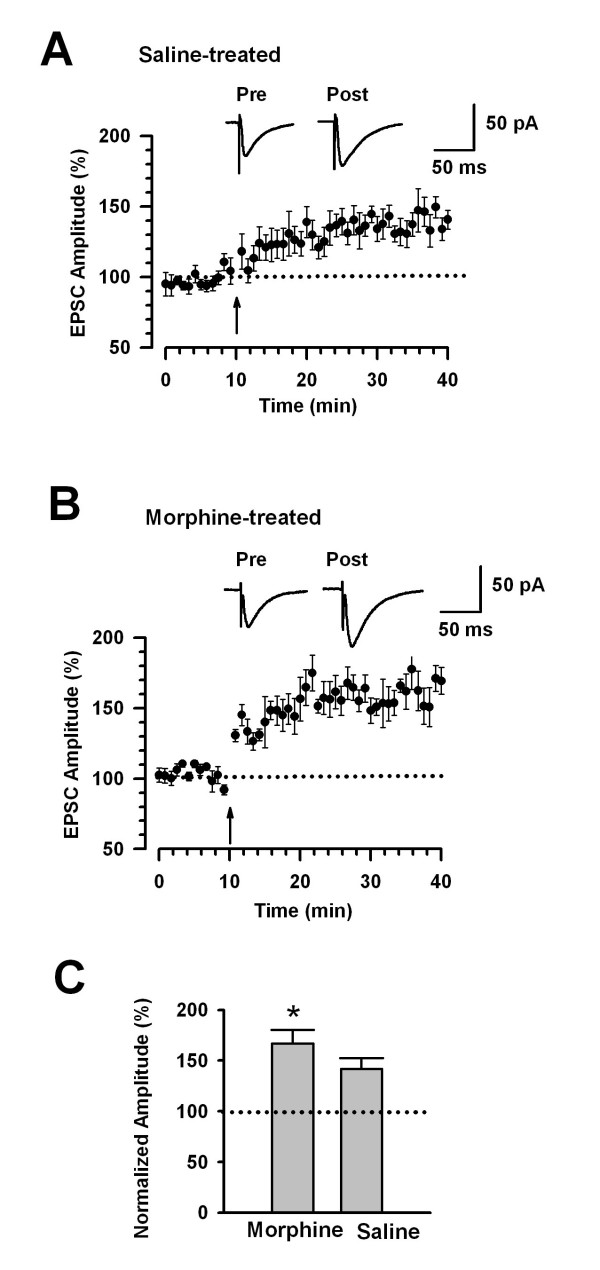
**Enhanced cingulate LTP in morphine treated mice**. (A) LTP was induced with the spike-timing protocol in ACC neurons in wild-type mice (n = 7). (B) Enhanced potentiation was observed in morphine treated mice (n = 8). The insets show averages of six EPSCs at baseline responses (1) and 30 min (2) after LTP induction (arrow). The dashed line indicates the mean basal synaptic response. (C) There was a significant increase in LTP in morphine treated mice compared to saline treated group.

In the ACC, NR2B receptors contribute to the induction of long-term potentiation (LTP) [20]. Since the function of NR2B was upregulated in the ACC of morphine treated mice, we investigated whether LTP was also enhanced. LTP was induced using the spike-timing protocol (see Materials and Methods) within 12 minutes after establishing the whole-cell configuration to avoid washout of intracellular contents that are critical for the establishment of synaptic plasticity. LTP was induced in the ACC of saline treated mice (141.7 ± 10.8% of baseline response, n = 7, p < 0.05 compared with baseline response before the stimulation, Figure 5A and 5C) after obtaining a stable baseline of AMPA receptor-mediated responses. Interestingly, the same protocol resulted in a significantly enhanced potentiation in morphine treated mice (166.9 ± 13.6% of baseline response, n = 8 slices; P < 0.05 compared with saline treated group, Figure 4B and 4C). These results suggest that chronic morphine treatment leads to the enhancement of LTP in the ACC.

## Discussion

In this study we demonstrate a role for the NMDA NR2B subunit in the expression and acquisition of analgesic tolerance and in responses to acute morphine analgesia. Additionally, we show that inhibiting NR2B receptors in the ACC can inhibit analgesic tolerance. Changes in synaptic plasticity associated with chronic drug use are well known, however, changes in prefrontal regions have not been documented. We show that morphine-tolerant animals not only show an increase in both the synaptic and extrasynaptic NR2B component of NMDA receptor responses but also exhibit facilitated LTP. This is the first study, to our knowledge, to look at the selective role of NMDA NR2B receptors in the expression and acquisition of opioid analgesic tolerance and in the expression of morphine-induced changes in synaptic plasticity in the ACC.

Although a myriad of studies have reported on the effects of NMDAR antagonists on morphine induced analgesia, the results are often conflicting and are probably due to different doses, experimental animals, time courses and behavioral tests used [26-28]. For example, sex differences in NMDAR antagonist induced modulation of morphine analgesia have been reported [6,28]. The direction of this modulation is also unclear, since some studies report that MK801 has no effect on morphine analgesia [29-31] while others report an inhibition [32,33] and others an increase [6,9]. The NMDAR antagonists LY 235959, dextromethorphan and AP5 also potentiate morphine analgesia [34-38].

The ability of NMDAR antagonists to reduce the development of tolerance has been extensively demonstrated [3]. MK801 attenuated the development of morphine tolerance and reduced withdrawal behaviors without affecting acute analgesia [8]. The NMDA receptor antagonists LY274614, dextromethorphan, AP5 and ketamine were all shown to attenuate analgesic tolerance [9,30,38-42]. Here we present evidence for a role of the NR2B subunit in development of analgesic tolerance. Although we are still unclear about the exact molecular mechanism for the NR2B containing NMDA receptor in morphine tolerance, one possible mechanism is that NR2B containing NMDA receptor may contribute to the induction of the synaptic process that may contribute to the establishment of morphine tolerance. Future studies are clearly needed to investigate this possibility. Since the use of most NMDAR antagonists is hindered by side-effects at clinically relevant doses, Ro 256981 may prove more useful in treating analgesic tolerance in patients.

Previous studies report a role for the NR2B subunit in acute morphine analgesia with conflicting results [6,43,44]. The NR2B antagonist ifenprodil was shown to both potentiated and prolong the analgesic effects of morphine at a dose that was itself antinociceptive [43]. A more recent study showed that a lower dose of ifenprodil was not antinociceptive but still potentiated and prolonged morphine analgesia [44]. A study reporting that Ro 256981 attenuated responses to morphine [6] seemingly contradicts our present findings. However, it is important to note that different doses and nociceptive tests were used. The present study used 5 mg/kg Ro 256981 and the hotplate test while Nemmani and colleagues (2004) used 50 mg/kg and a hot water tail flick paradigm. Importantly, the dose of Ro 256981 used in the present study was previously shown not to affect motor coordination, acute thermal and mechanical sensitivity [21].

Human brain imaging shows that the ACC is strongly activated when subjects are exposed to drug-associated cues [45-47]. For example, the ACC is activated during cocaine use and also during cue-induced craving for cocaine [48,49]. While lesion of the rat ACC did not affect cocaine self-administration, it was effective at preventing drug associated cues from inducing cocaine seeking behavior [50]. Furthermore, when rats were exposed to an environment where they were previously allowed to self administer cocaine, immediate early gene expression, indicative of neuronal activity, was increased in the ACC [51]. Neuronal activity was also increased in the ACC after exposure to a morphine-paired environment [52,53]. The mu opioid receptor is robustly expressed in the ACC [54] and microinjection of morphine directly into the ACC reduced the affective component of pain [55]. Acute and chronic morphine treatment reduced the extracellular levels of glutamate in the ACC [56], suggesting that glutamatergic systems in the ACC may play a role in morphine's effects. Further studies are needed to identify the precise role that the ACC plays in the molecular and behavioral adaptations that occur with the chronic use of drugs of abuse.

Although the relationship between NMDA receptors and the effects of morphine has been thoroughly investigated, fewer studies examine changes in NMDA receptor mediated responses after prolonged morphine use. Chronic morphine treatment enhanced NMDA receptor mediated responses in spinal cord dorsal horn neurons, however the individual contribution of NR2A or NR2B subunits was not addressed [57]. In the present study, we report that chronic morphine treatment produced significant changes in the NMDA receptor mediated responses. These findings indicate that chronic morphine treatment may trigger long-term upregulation of NMDA receptors along the somatosensory pathways and pain perception related cortical areas. Our electrophysiological data indicate that alterations in NR2B receptor function may not only occur at synaptic sites, but also at extrasynaptic areas. This finding is consistent with recent evidence suggesting that NR2B subunits are located at extrasynaptic suites, and are recruited when repetitive stimulation allows for the spillover of glutamate [25]. Further study is needed to uncover the molecular signaling pathways that are engaged when chronic morphine treatment leads to the modulation of NR2B receptor mediated responses.

Recent studies show that NR2B receptor activation contributes, at least in part, to the induction of synaptic LTP in the ACC [20]. We found that LTP was significantly enhanced in the ACC of morphine tolerant mice compared to saline treated animals, suggesting that NR2B receptor mediated responses were enhanced after chronic morphine treatment. We cannot rule out the possible contribution of other signaling molecules such as cAMP pathways and other messengers that are downstream from opiate receptors.

## Conclusion

In summary, we present evidence for a role of the NMDA subunit NR2B in morphine analgesia and opioid tolerance. We also show that inhibiting NR2B receptors in the ACC can inhibit behavioral responses in tolerant animals, suggesting for the first time that this prefrontal region plays a role in opioid tolerance. We have previously shown that NMDA NR2B receptors in the ACC play a role in persistent pain, memory and LTP [58]. Our results support the idea that the alterations that occur in response to drugs of abuse are mirrored by the mechanisms that establish learning and memory and demonstrate a novel role for the ACC in the expression of opioid analgesic tolerance. Our results suggest that drugs targeting the NR2B subunit may prove useful in attenuating analgesic tolerance to morphine that occurs clinically.

## Methods

### Animals

Adult male (8–10 weeks old) C57Bl/6 mice from Charles River were used in all experiments. At the conclusion of experiments, animals were humanely killed by an overdose of inhaled anesthetic (isoflurane). The animals were housed on a 12 h: 12 h light: dark cycle with food and water available *ad libitum*. All mouse protocols are in accordance with NIH guidelines and were approved by the Animal Care and Use Committee at the University of Toronto. All experiments were performed blind to the treatment.

### Hot-plate test

The hotplate consists of a thermally-controlled metal plate (55°C), surrounded by four Plexiglas walls (Columbia Instruments; Columbus, Ohio). The time between placement of the animal on the plate and the licking or lifting of a hindpaw is measured with a digital timer. Mice are removed from the hotplate immediately after the first response and a cut-off time of 30 seconds was imposed to prevent tissue damage. Response latencies are reported as a percentage of maximal possible effect (MPE) [(response latency-baseline response latency)/(cut off latency-baseline response latency)*100].

### Morphine administration

To test for acute morphine induced antinociception, Ro 256981 (Tocris Cookson, St Louis, MO, 5 mg/kg in saline) or an equivalent volume of saline was injected 10–15 minutes before morphine (10 mg/kg s.c.). To study the role of NR2B in the expression of analgesic tolerance, mice were injected once a day for eight days with 10 mg/kg morphine (s.c.) and received Ro 256981 (5 mg/kg, i.p.) before the last morphine injection. To test for a role in the acquisition of tolerance, mice were injected with Ro 256981 (5 mg/kg, i.p.) before morphine (10 mg/kg s.c.) daily for eight days.

### ACC cannula implantation and microinjection

Under ketamine and xylazine anesthesia, 24-gauge guide cannulas were implanted bilaterally into the ACC (0.7 mm anterior to Bregma, ± 0.4 mm lateral from the midline, 1.7 mm beneath the surface of the skull). Mice were given at least 2 weeks to recover after cannula implantation. For intra-ACC injections, mice were anesthetized with 2–3% isoflurane anesthesia in a gas mixture of 30% O_2 _balanced with nitrogen and placed in a Kopf stereotaxic instrument. A 30-gauge injection cannula was 0.1 mm lower than the guide. The microinjection apparatus consisted of a Hamilton syringe (5 ml), connected to an injector needle (30 gauge) by a thin polyethylene tube, and motorized syringe pump. Ro 256981 (1.0 μg/μl, in saline) was infused into each side of the ACC at a rate of 0.05 μl/min, an equivalent volume of saline was used as a control. After injection, the microinjection needle was left in place for at least 5 minutes.

### Whole-cell Patch Clamp Recordings

Mice were anesthetized with 1–2% isoflurane before decapitation. Coronal slices containing the ACC (300 μm) were prepared using methods reported previously [21]. Slices were then transferred to a room temperature submerged recovery chamber with oxygenated (95% O_2 _and 5% CO_2_) solution containing (in mM): NaCl, 124; NaHCO_3_, 25; KCl, 2.5; KH_2_PO_4_, 1; CaCl_2_, 2; MgSO_4_, 2; glucose, 10. After a one-hour recovery period, slices were placed in a recording chamber on the stage of an Axioskop 2FS microscope (Zeiss, Thornwood, NY) equipped with infrared DIC optics for patch clamp recordings. Postsynaptic currents were recorded with an Axon 200B amplifier (Molecular Devices, Union City, CA). Excitatory postsynaptic currents (EPSCs) were recorded from layer II/III neurons with an Axon 200B amplifier (Molecular devices, CA) and stimuli were delivered by a bipolar tungsten stimulating electrode placed in layer V of ACC slices. Electric square-wave voltage pulses (200 μs, 4–14 V) were generated using a Grass S88 stimulator (Grass instrument Co., Quincy, MA) attached to a Grass SIU5D isolator unit. EPSCs were induced by repetitive stimulations at 0.02 Hz and neurons were voltage clamped at -70 mV. The recording pipettes (3–5 MΩ) were filled with a solution containing (mM): 145 K-gluconate, 5 NaCl, 1 MgCl_2_, 0.2 EGTA, 10 HEPES, 2 Mg-ATP, and 0.1 Na_3_-GTP (adjusted to pH 7.2 with KOH). Unless stated otherwise, the membrane potential was held at -65 mV throughout all experiments.

LTP was induced within 12 min after establishing the whole-cell configuration to prevent a wash out effect [59]. The LTP induction protocol used (referred to as spike-timing protocol) pairing 3 presynaptic stimuli, which caused 3 EPSPs (10 ms ahead), with 3 postsynaptic APs at 30 Hz, paired 15 times every 5 s [20]. The NMDA receptor-mediated component of EPSCs was pharmacologically isolated in ACSF containing: CNQX (20 μM) and picrotoxin (100 μM). The patch electrodes for NMDA receptor-mediated EPSCs contained (in mM) 102 cesium gluconate, 5 TEA chloride, 3.7 NaCl, 11 BAPTA, 0.2 EGTA, 20 HEPES, 2 MgATP, 0.3 NaGTP, and 5 QX-314 chloride (adjusted to pH 7.2 with CsOH). Neurons were voltage clamped at -20 mV and NMDA receptor-mediated EPSCs were evoked at 0.05 Hz. Access resistance was 15–30 MΩ and was monitored throughout the experiment. Data were discarded if access resistance changed more than 15% during an experiment. Statistical comparisons were performed using the Student's t-test.

### Data analysis and statistics

Statistical comparisons were made using one way or two way repeated measures ANOVA (Student-Newmann-Keuls test was used for *post hoc *comparison). All data is represented by the mean +/- S.E.M. In all cases, p < 0.05 is considered statistically significant.

## Authors' contributions

SK, FS and JQ performed the behavioral experiments, LJW carried out the electrophysiological experiments, SK and WLJ performed the statistical analysis, SK, LJW and MZ designed the study and wrote the manuscript. All authors read and approved the final manuscript.
